# Chlorido(2-imino­methyl-3-fluoro­phenyl-κ^2^ *C* ^1^,*N*)tris­(trimethyl­phos­phane-κ*P*)iron

**DOI:** 10.1107/S1600536811015030

**Published:** 2011-05-07

**Authors:** Xiaofeng Xu, Xiaoyan Li

**Affiliations:** a School of Chemistry and Chemical Engineering, Shandong University, Jinan 250100, People’s Republic of China

## Abstract

The title compound, [Fe(C_7_H_5_FN)Cl(C_3_H_9_P)_3_], was obtained as a product of the reaction of [Fe(Me_3_P)_4_] with a molar equivalent of (2-chloro-6-fluoro­phen­yl)methanimine in diethyl ether. This compound is sensitive to air, and rapidly decomposes when exposed to air for a few minutes. The Fe atom has an octa­hedral coordination geometry in which the bidentate fluoro­phenyl methanimine ligand forms the equatorial plane with the Cl atom and one of the trimethyl­phosphane ligands. The other two trimethyl­phosphane ligands are located in the axial positions. In the crystal, an N—H⋯Cl hydrogen bond occurs.

## Related literature

For related literature regarding C—Cl bond activation, see: Wang *et al.* (2007 ▶); Wang & Love (2008 ▶); Shi *et al.* (2009 ▶). Related crystal structures of iron compounds have not yet been reported in the literature. For substituted phenyl­methanimine coordinated dihydride complexes of osmium, see: Schloerer *et al.* (2006 ▶); Barea *et al.* (1998 ▶).
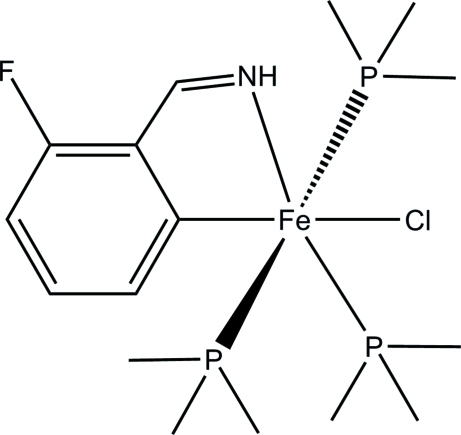


## Experimental

### 

#### Crystal data

[Fe(C_7_H_5_FN)Cl(C_3_H_9_P)_3_]
*M* *_r_* = 441.64
Monoclinic, 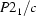

*a* = 8.9879 (6) Å
*b* = 19.4457 (13) Å
*c* = 13.5438 (7) Å
β = 114.937 (3)°
*V* = 2146.4 (2) Å^3^
*Z* = 4
Mo *K*α radiation
μ = 1.06 mm^−1^
*T* = 298 K
0.20 × 0.18 × 0.15 mm

#### Data collection

Bruker APEXII CCD diffractometer
Absorption correction: multi-scan (*SADABS*; Bruker, 2001 ▶) *T*_min_ = 0.816, *T*_max_ = 0.858
12499 measured reflections
4859 independent reflections
4109 reflections with *I* > 2σ(*I*)
*R*_int_ = 0.024

#### Refinement

*R*[*F* ^2^ > 2σ(*F* ^2^)] = 0.029
*wR*(*F* ^2^) = 0.071
*S* = 1.04
4859 reflections
217 parameters
H-atom parameters constrained
Δρ_max_ = 0.37 e Å^−3^
Δρ_min_ = −0.23 e Å^−3^

### 

Data collection: *APEX2* (Bruker, 2004 ▶); cell refinement: *SAINT-Plus* (Bruker, 2001 ▶); data reduction: *SAINT-Plus*; program(s) used to solve structure: *SHELXS97* (Sheldrick, 2008 ▶); program(s) used to refine structure: *SHELXL97* (Sheldrick, 2008 ▶); molecular graphics: *SHELXTL* (Sheldrick, 2008 ▶); software used to prepare material for publication: *SHELXTL*.

## Supplementary Material

Crystal structure: contains datablocks global, I. DOI: 10.1107/S1600536811015030/ez2239sup1.cif

Structure factors: contains datablocks I. DOI: 10.1107/S1600536811015030/ez2239Isup2.hkl

Additional supplementary materials:  crystallographic information; 3D view; checkCIF report

## Figures and Tables

**Table 1:** Hydrogen-bond geometry (Å, °)

*D*—H⋯*A*	*D*—H	H⋯*A*	*D*⋯*A*	*D*—H⋯*A*
N1—H1⋯Cl1^i^	0.86	2.53	3.3339 (15)	157

Symmetry code: (i) 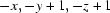.

**Fig. 1.**
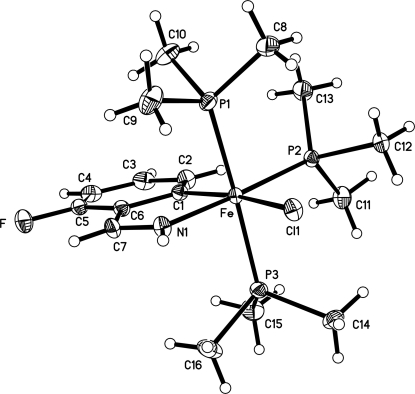
A view of the structure of (I), showing the atomic numbering scheme and 30% probability displacement ellipsoids.

## supplementary crystallographic information

### Comment

C-Cl bond activation and C-C coupling reactions are of great interest, with much focus on ortho-substitued imines (Wang *et al.*, 2007; Wang & Love, 2008), where Pt complexes have been used to catalyze the cross coupling of imines and dimethylzinc reagents through C-X bond activation. We have previously shown that the iron complexes supported by trimethylphosphine can easily activate the C-X (X=Cl,F) bond (Shi *et al.*, 2009).

Reaction of the low valent complex of Fe(PMe_3_)_4_ with (2-chloro-6-fluorophenyl)methanimine afforded the title compound. The *ortho* C—Cl bond was selectively activated due to its energy being lower than the C—F bond. The coordination of the imine group and one of the C atoms led to the formation of a five membered chelate ring which can provide enough energy to actiatve the C—Cl bond. It indicates that with the assistance of the imine group iron(0) complexes can easily activate the C—Cl bond affording iron(II) chloride complexes. Subsequently, C—C coupling reactions with organometallic reagents may occur.

In the title molecule (Fig. 1) the iron atom lies in an octahedral geometry in which atoms C1, N1, Cl1 and P2 form the basal plane with P1 and P3 in the axial positions. The two axial groups are located in a distorted linear geometry. A five-membered chelate ring is formed by C1, C6, C7, N1 and Fe. The bite angle of N1—Fe—C1 is 81.08 (7)°. The sum of internal bond angles (360.0 °) of N1—Fe—C1, C1—Fe—P2, P2—Fe—Cl1 and Cl1—Fe—N1 indicates nearly perfect planarity. The N—H···Cl interaction is rather short (2.53 Å). The probable reason is the steric effect of atom P2 which forces the Cl1 atom closer to the H1 atom. The C1—Fe—Cl1 angle is 167.34 (5)°, while the N1—Fe—Cl1 angle (86.27 (4)°) is less than 90°. Related structures of Os complexes containing the same iminomethyl ligand have been reported in the literature (Barea *et al.*, 1998; Schloerer *et al.*, 2006), although one molecule of hydrogen occupies the position *trans* to the nitrogen atom instead of one of the trimethylphosphine ligands. In this structure the N—Os—Cl angle is also less than 90°, and also contains a similar short N—H···Cl interaction.

### Experimental

A sample of Fe(PMe_3_)_4_ (0.50 g, 1.39 mmol) in 30 ml of diethyl ether was combined with a solution of (2-chloro-6-fluorophenyl)methanimine (0.22 g, 1.39 mmol) in diethyl ether (20 ml) at -80 °. The reaction mixture was warmed to ambient temperature and stirred for 24 h to form a red solution. The volatiles were removed *in vacuo*, and the resulting solid was extracted with pentane (40 ml). Crystallization at -15 ° afforded red crystals suitable for X-ray diffraction analysis (yield 0.40 g, 65%), dec. > 86 °.

### Refinement

All H atoms on C were placed in calculated positions with a C—H bond distance of 0.93 or 0.96 Å and *U*_iso_(H) = 1.2*U*_eq_ of the carrier atom.

### Crystal data

**Table:**

[Fe(C_7_H_5_FN)Cl(C_3_H_9_P)_3_]	*F*(000) = 928
*M**_r_* = 441.64	*D*_x_ = 1.367 Mg m^−^^3^
Monoclinic, *P*2_1_/*c*	Mo *K*α radiation, λ = 0.71073 Å
Hall symbol: -P2ybc	Cell parameters from 5013 reflections
*a* = 8.9879 (6) Å	θ = 2.5–27.5°
*b* = 19.4457 (13) Å	µ = 1.06 mm^−^^1^
*c* = 13.5438 (7) Å	*T* = 298 K
β = 114.937 (3)°	Block, red
*V* = 2146.4 (2) Å^3^	0.20 × 0.18 × 0.15 mm
*Z* = 4	

### Data collection

**Table:**

Bruker APEXII CCD diffractometer	4859 independent reflections
Radiation source: fine-focus sealed tube	4109 reflections with *I* > 2σ(*I*)
graphite	*R*_int_ = 0.024
φ and ω scans	θ_max_ = 27.5°, θ_min_ = 2.0°
Absorption correction: multi-scan (*SADABS*; Bruker, 2001)	*h* = −10→11
*T*_min_ = 0.816, *T*_max_ = 0.858	*k* = −24→25
12499 measured reflections	*l* = −17→13

### Refinement

**Table:**

Refinement on *F*^2^	Primary atom site location: structure-invariant direct methods
Least-squares matrix: full	Secondary atom site location: difference Fourier map
*R*[*F*^2^ > 2σ(*F*^2^)] = 0.029	Hydrogen site location: inferred from neighbouring sites
*wR*(*F*^2^) = 0.071	H-atom parameters constrained
*S* = 1.04	*w* = 1/[σ^2^(*F*_o_^2^) + (0.0334*P*)^2^ + 0.4204*P*] where *P* = (*F*_o_^2^ + 2*F*_c_^2^)/3
4859 reflections	(Δ/σ)_max_ = 0.001
217 parameters	Δρ_max_ = 0.37 e Å^−^^3^
0 restraints	Δρ_min_ = −0.23 e Å^−^^3^

### Special details

**Table:**

Geometry. All e.s.d.'s (except the e.s.d. in the dihedral angle between two l.s. planes) are estimated using the full covariance matrix. The cell e.s.d.'s are taken into account individually in the estimation of e.s.d.'s in distances, angles and torsion angles; correlations between e.s.d.'s in cell parameters are only used when they are defined by crystal symmetry. An approximate (isotropic) treatment of cell e.s.d.'s is used for estimating e.s.d.'s involving l.s. planes.
Refinement. Refinement of *F*^2^ against ALL reflections. The weighted *R*-factor *wR* and goodness of fit *S* are based on *F*^2^, conventional *R*-factors *R* are based on *F*, with *F* set to zero for negative *F*^2^. The threshold expression of *F*^2^ > σ(*F*^2^) is used only for calculating *R*-factors(gt) *etc*. and is not relevant to the choice of reflections for refinement. *R*-factors based on *F*^2^ are statistically about twice as large as those based on *F*, and *R*- factors based on ALL data will be even larger.

### Fractional atomic coordinates and isotropic or equivalent isotropic displacement parameters (Å^2^)

**Table:**

	*x*	*y*	*z*	*U*_iso_*/*U*_eq_	
Fe	0.02988 (3)	0.391645 (11)	0.325636 (18)	0.01883 (7)	
P3	0.25510 (6)	0.35114 (2)	0.46464 (4)	0.02733 (11)	
P2	0.14694 (5)	0.39138 (2)	0.20985 (4)	0.02377 (11)	
P1	−0.19959 (5)	0.44130 (2)	0.20154 (4)	0.02855 (11)	
C1	−0.0641 (2)	0.29926 (8)	0.29408 (13)	0.0239 (3)	
C6	−0.1656 (2)	0.28469 (9)	0.34912 (13)	0.0249 (4)	
C5	−0.2479 (2)	0.22233 (10)	0.33495 (15)	0.0311 (4)	
C4	−0.2322 (2)	0.17074 (10)	0.27239 (15)	0.0377 (5)	
H4	−0.2877	0.1293	0.2647	0.045*	
C3	−0.1304 (3)	0.18198 (9)	0.22048 (15)	0.0372 (5)	
H3	−0.1154	0.1472	0.1784	0.045*	
C2	−0.0503 (2)	0.24454 (9)	0.23040 (15)	0.0319 (4)	
H2	0.0153	0.2505	0.1933	0.038*	
N1	−0.08235 (18)	0.39126 (7)	0.42146 (12)	0.0272 (3)	
H1	−0.0735	0.4256	0.4636	0.033*	
C7	−0.1695 (2)	0.33880 (9)	0.42040 (14)	0.0291 (4)	
H7	−0.2296	0.3360	0.4618	0.035*	
C12	0.2364 (2)	0.47338 (9)	0.19708 (15)	0.0320 (4)	
H12A	0.3218	0.4860	0.2663	0.048*	
H12B	0.1530	0.5082	0.1737	0.048*	
H12C	0.2816	0.4690	0.1445	0.048*	
C13	0.0237 (3)	0.37148 (12)	0.06515 (15)	0.0414 (5)	
H13A	−0.0624	0.4048	0.0344	0.062*	
H13B	−0.0233	0.3265	0.0587	0.062*	
H13C	0.0925	0.3728	0.0269	0.062*	
C11	0.3211 (2)	0.33487 (10)	0.22908 (17)	0.0375 (5)	
H11A	0.3555	0.3428	0.1719	0.056*	
H11B	0.2884	0.2877	0.2273	0.056*	
H11C	0.4105	0.3445	0.2981	0.056*	
C8	−0.1846 (3)	0.51567 (12)	0.1234 (2)	0.0500 (6)	
H8A	−0.2926	0.5328	0.0792	0.075*	
H8B	−0.1322	0.5022	0.0776	0.075*	
H8C	−0.1211	0.5511	0.1725	0.075*	
C10	−0.3503 (2)	0.38749 (12)	0.09655 (18)	0.0475 (6)	
H10A	−0.3822	0.3504	0.1302	0.071*	
H10B	−0.3030	0.3692	0.0504	0.071*	
H10C	−0.4449	0.4146	0.0538	0.071*	
C9	−0.3291 (3)	0.47893 (11)	0.2609 (2)	0.0479 (6)	
H9A	−0.4121	0.5070	0.2075	0.072*	
H9B	−0.2632	0.5067	0.3224	0.072*	
H9C	−0.3803	0.4429	0.2840	0.072*	
C14	0.4433 (2)	0.40139 (11)	0.50640 (17)	0.0441 (5)	
H14A	0.5319	0.3771	0.5623	0.066*	
H14B	0.4286	0.4451	0.5340	0.066*	
H14C	0.4684	0.4084	0.4449	0.066*	
C15	0.3272 (3)	0.26346 (10)	0.46240 (18)	0.0451 (5)	
H15A	0.3641	0.2595	0.4057	0.068*	
H15B	0.2391	0.2317	0.4491	0.068*	
H15C	0.4163	0.2532	0.5313	0.068*	
C16	0.2353 (3)	0.34609 (12)	0.59360 (16)	0.0458 (5)	
H16A	0.3393	0.3340	0.6511	0.069*	
H16B	0.1554	0.3117	0.5881	0.069*	
H16C	0.2008	0.3899	0.6092	0.069*	
F	−0.34624 (14)	0.21266 (6)	0.38775 (10)	0.0473 (3)	
Cl1	0.11769 (5)	0.50640 (2)	0.39209 (4)	0.02803 (10)	

### Atomic displacement parameters (Å^2^)

**Table:**

	*U*^11^	*U*^22^	*U*^33^	*U*^12^	*U*^13^	*U*^23^
Fe	0.02089 (13)	0.01713 (12)	0.02020 (13)	−0.00157 (9)	0.01036 (10)	−0.00253 (9)
P3	0.0284 (2)	0.0237 (2)	0.0260 (2)	−0.00102 (18)	0.00766 (19)	0.00456 (18)
P2	0.0242 (2)	0.0271 (2)	0.0231 (2)	−0.00228 (18)	0.01291 (18)	−0.00302 (17)
P1	0.0204 (2)	0.0289 (2)	0.0338 (3)	0.00095 (18)	0.00892 (19)	0.00162 (19)
C1	0.0254 (8)	0.0222 (8)	0.0227 (8)	−0.0012 (7)	0.0089 (7)	−0.0011 (7)
C6	0.0263 (9)	0.0237 (8)	0.0238 (8)	−0.0022 (7)	0.0096 (7)	0.0012 (7)
C5	0.0281 (9)	0.0313 (10)	0.0288 (9)	−0.0073 (8)	0.0069 (8)	0.0074 (8)
C4	0.0423 (11)	0.0239 (9)	0.0336 (10)	−0.0110 (8)	0.0031 (9)	0.0016 (8)
C3	0.0509 (12)	0.0219 (9)	0.0315 (10)	−0.0031 (8)	0.0103 (9)	−0.0076 (8)
C2	0.0395 (10)	0.0256 (9)	0.0329 (10)	−0.0050 (8)	0.0175 (8)	−0.0085 (8)
N1	0.0347 (8)	0.0233 (7)	0.0299 (8)	−0.0039 (6)	0.0198 (7)	−0.0072 (6)
C7	0.0325 (10)	0.0324 (10)	0.0290 (9)	−0.0031 (8)	0.0194 (8)	−0.0001 (8)
C12	0.0320 (10)	0.0350 (10)	0.0337 (10)	−0.0038 (8)	0.0184 (8)	0.0030 (8)
C13	0.0455 (12)	0.0559 (13)	0.0254 (10)	−0.0109 (10)	0.0174 (9)	−0.0086 (9)
C11	0.0375 (11)	0.0365 (11)	0.0479 (12)	0.0022 (9)	0.0273 (10)	−0.0050 (9)
C8	0.0326 (11)	0.0499 (13)	0.0586 (14)	0.0082 (10)	0.0106 (10)	0.0259 (11)
C10	0.0277 (10)	0.0572 (14)	0.0437 (13)	−0.0041 (10)	0.0015 (9)	−0.0069 (10)
C9	0.0316 (11)	0.0448 (12)	0.0696 (16)	0.0098 (9)	0.0235 (11)	−0.0042 (11)
C14	0.0296 (10)	0.0465 (12)	0.0406 (12)	−0.0066 (9)	−0.0004 (9)	0.0105 (10)
C15	0.0475 (12)	0.0315 (11)	0.0546 (13)	0.0139 (9)	0.0197 (11)	0.0145 (10)
C16	0.0600 (14)	0.0450 (12)	0.0273 (10)	−0.0034 (11)	0.0135 (10)	0.0072 (9)
F	0.0466 (7)	0.0466 (7)	0.0524 (7)	−0.0150 (6)	0.0245 (6)	0.0088 (6)
Cl1	0.0366 (2)	0.01904 (19)	0.0327 (2)	−0.00468 (17)	0.01878 (19)	−0.00525 (17)

### Geometric parameters (Å, °)

**Table:**

Fe—N1	1.9508 (14)	C7—H7	0.9300
Fe—C1	1.9544 (16)	C12—H12A	0.9600
Fe—P2	2.2265 (5)	C12—H12B	0.9600
Fe—P3	2.2470 (5)	C12—H12C	0.9600
Fe—P1	2.2556 (5)	C13—H13A	0.9600
Fe—Cl1	2.4111 (5)	C13—H13B	0.9600
P3—C14	1.825 (2)	C13—H13C	0.9600
P3—C15	1.829 (2)	C11—H11A	0.9600
P3—C16	1.832 (2)	C11—H11B	0.9600
P2—C12	1.8268 (18)	C11—H11C	0.9600
P2—C11	1.8393 (19)	C8—H8A	0.9600
P2—C13	1.8404 (19)	C8—H8B	0.9600
P1—C10	1.824 (2)	C8—H8C	0.9600
P1—C9	1.824 (2)	C10—H10A	0.9600
P1—C8	1.830 (2)	C10—H10B	0.9600
C1—C2	1.408 (2)	C10—H10C	0.9600
C1—C6	1.429 (2)	C9—H9A	0.9600
C6—C5	1.391 (2)	C9—H9B	0.9600
C6—C7	1.439 (2)	C9—H9C	0.9600
C5—C4	1.359 (3)	C14—H14A	0.9600
C5—F	1.364 (2)	C14—H14B	0.9600
C4—C3	1.386 (3)	C14—H14C	0.9600
C4—H4	0.9300	C15—H15A	0.9600
C3—C2	1.391 (3)	C15—H15B	0.9600
C3—H3	0.9300	C15—H15C	0.9600
C2—H2	0.9300	C16—H16A	0.9600
N1—C7	1.283 (2)	C16—H16B	0.9600
N1—H1	0.8600	C16—H16C	0.9600
			
N1—Fe—C1	81.08 (7)	C6—C7—H7	123.2
N1—Fe—P2	177.39 (5)	P2—C12—H12A	109.5
C1—Fe—P2	97.64 (5)	P2—C12—H12B	109.5
N1—Fe—P3	88.69 (5)	H12A—C12—H12B	109.5
C1—Fe—P3	90.84 (5)	P2—C12—H12C	109.5
P2—Fe—P3	93.61 (2)	H12A—C12—H12C	109.5
N1—Fe—P1	86.02 (5)	H12B—C12—H12C	109.5
C1—Fe—P1	93.18 (5)	P2—C13—H13A	109.5
P2—Fe—P1	91.794 (19)	P2—C13—H13B	109.5
P3—Fe—P1	172.79 (2)	H13A—C13—H13B	109.5
N1—Fe—Cl1	86.27 (4)	P2—C13—H13C	109.5
C1—Fe—Cl1	167.34 (5)	H13A—C13—H13C	109.5
P2—Fe—Cl1	95.022 (17)	H13B—C13—H13C	109.5
P3—Fe—Cl1	88.522 (18)	P2—C11—H11A	109.5
P1—Fe—Cl1	86.246 (18)	P2—C11—H11B	109.5
C14—P3—C15	102.46 (10)	H11A—C11—H11B	109.5
C14—P3—C16	100.56 (11)	P2—C11—H11C	109.5
C15—P3—C16	98.25 (10)	H11A—C11—H11C	109.5
C14—P3—Fe	117.51 (7)	H11B—C11—H11C	109.5
C15—P3—Fe	120.89 (7)	P1—C8—H8A	109.5
C16—P3—Fe	113.72 (8)	P1—C8—H8B	109.5
C12—P2—C11	98.76 (9)	H8A—C8—H8B	109.5
C12—P2—C13	100.02 (9)	P1—C8—H8C	109.5
C11—P2—C13	96.86 (10)	H8A—C8—H8C	109.5
C12—P2—Fe	114.99 (6)	H8B—C8—H8C	109.5
C11—P2—Fe	121.91 (7)	P1—C10—H10A	109.5
C13—P2—Fe	119.97 (7)	P1—C10—H10B	109.5
C10—P1—C9	99.88 (11)	H10A—C10—H10B	109.5
C10—P1—C8	102.37 (11)	P1—C10—H10C	109.5
C9—P1—C8	98.87 (11)	H10A—C10—H10C	109.5
C10—P1—Fe	118.72 (8)	H10B—C10—H10C	109.5
C9—P1—Fe	113.34 (8)	P1—C9—H9A	109.5
C8—P1—Fe	120.13 (7)	P1—C9—H9B	109.5
C2—C1—C6	114.22 (15)	H9A—C9—H9B	109.5
C2—C1—Fe	133.41 (14)	P1—C9—H9C	109.5
C6—C1—Fe	112.36 (12)	H9A—C9—H9C	109.5
C5—C6—C1	121.36 (16)	H9B—C9—H9C	109.5
C5—C6—C7	124.61 (16)	P3—C14—H14A	109.5
C1—C6—C7	114.01 (15)	P3—C14—H14B	109.5
C4—C5—F	119.09 (16)	H14A—C14—H14B	109.5
C4—C5—C6	122.71 (18)	P3—C14—H14C	109.5
F—C5—C6	118.19 (17)	H14A—C14—H14C	109.5
C5—C4—C3	117.68 (17)	H14B—C14—H14C	109.5
C5—C4—H4	121.2	P3—C15—H15A	109.5
C3—C4—H4	121.2	P3—C15—H15B	109.5
C4—C3—C2	120.94 (18)	H15A—C15—H15B	109.5
C4—C3—H3	119.5	P3—C15—H15C	109.5
C2—C3—H3	119.5	H15A—C15—H15C	109.5
C3—C2—C1	123.01 (18)	H15B—C15—H15C	109.5
C3—C2—H2	118.5	P3—C16—H16A	109.5
C1—C2—H2	118.5	P3—C16—H16B	109.5
C7—N1—Fe	118.71 (12)	H16A—C16—H16B	109.5
C7—N1—H1	120.6	P3—C16—H16C	109.5
Fe—N1—H1	120.6	H16A—C16—H16C	109.5
N1—C7—C6	113.67 (15)	H16B—C16—H16C	109.5
N1—C7—H7	123.2		

### Hydrogen-bond geometry (Å, °)

**Table:**

*D*—H···*A*	*D*—H	H···*A*	*D*···*A*	*D*—H···*A*
N1—H1···Cl1^i^	0.86	2.53	3.3339 (15)	157.

Symmetry codes: (i) −*x*, −*y*+1, −*z*+1.
